# Perineal stapled prolapse resection in combination with Thiersch operation for relapsed rectal prolapse: a case report

**DOI:** 10.1186/s40792-021-01287-4

**Published:** 2021-09-03

**Authors:** Teppei Kamada, Hironori Ohdaira, Junji Takahashi, Yoshinobu Fuse, Wataru Kai, Keigo Nakashima, Yuichi Nakaseko, Norihiko Suzuki, Masashi Yoshida, Takeo Usui, Yutaka Suzuki

**Affiliations:** 1 Department of Surgery, International University of Health and Welfare Hospital, 537-3, Iguchi, Nasushiobara, Tochigi 329-2763 Japan; 2 Department of Orthopedics, Nasu Central Hospital, 1453, Shimoishigami, Otawara, Tochigi 324-0036 Japan

**Keywords:** High-risk surgery, Perineal stapled prolapse resection, Rectal prolapse, Surgical repair, Thiersch operation

## Abstract

**Background:**

Treatment options for complete rectal prolapse include over 100 procedures. In previous reports, operative rectal prolapse repair, regardless of the technique by perineal approach, was associated with high recurrence rates. However, there is no consensus on the optimal surgical procedure for relapsed rectal prolapse.

**Case presentation:**

A 97-year-old woman was admitted to our hospital with a chief complaint of complete rectal prolapse measuring > 5 cm. The patient had a history of laparoscopic anterior suture rectopexy without sigmoid resection under general anesthesia for complete rectal prolapse one year prior. The patient’s postoperative course was uneventful. However, her dementia worsened (Hasegawa’s dementia scale: 5/30 points) after the first operation. Further, moderate-to-severe aortic valve stenosis was first diagnosed with heart failure 6 months after the operation. Nine months after the initial surgery, she experienced a recurrence of complete rectal prolapse measuring approximately 5 cm. Considering the coexistence of advanced age, severe dementia, and aortic valve stenosis, surgery under general anesthesia was not indicated. Perineal stapled prolapse resection in combination with the t operation was planned because of its minimal invasiveness and shortened hospital stay. The procedure was performed by a team of two surgeons in the jack knife position, under spinal anesthesia.

The prolapse was cut along the long-axis direction with three linear staplers and resected along the short-axis direction with four linear staplers. The cross-section of the linear stapler was reinforced with 3-0 Vicryl sutures. After rectal resection, the Thiersch operation using 1-0 nylon thread 1 cm away from the anal verge was additionally performed. The operative time was 24 min, and intraoperative blood loss was 1 mL. The postoperative course was uneventful. Three months after the operation, no recurrence was observed, and defecation function was good with improvements of Wexner score.

**Conclusions:**

Perineal stapled prolapse resection in combination with the Thiersch operation could be a useful option for patients with relapsed rectal prolapse and with poor general condition, who are not indicated for other surgical procedures.

## Background

Patients with complete rectal prolapse are often impaired regarding surgical tolerance because a large number of these patients are elderly and have high risks from underlying diseases [1, 2]. Therefore, treatment plans require both curative and palliative options.

Surgery for complete rectal prolapse is roughly divided into abdominal and perineal procedures. According to the American Society for Colon and Rectal Surgeons guidelines [3], surgical treatment should be tailored to a patient’s comorbidities, age, and bowel function and the surgeon’s preference and experience. There is no consensus regarding which procedure is better.

In general, when a patient has low surgical tolerance, a perineal operation is often performed, but the recurrence rate is high. Conversely, when a patient’s surgical tolerance is sufficient, laparotomy or a laparoscopic abdominal operation is performed, but this requires a longer operation time, is more invasive, and has a higher complication rate than perineal operations. Although operative rectal prolapse repair has been associated with high recurrence rates in previous reports [3], there have been no recommended surgical procedures for relapsed rectal prolapse. We report a favorable course of relapsed rectal prolapse with successful perineal stapled prolapse resection in combination with the Thiersch operation in a 97-year old super-elderly patient after anterior suture rectopexy without sigmoid resection.

## Case presentation

A 97-year-old woman was admitted to our hospital with a chief complaint of complete rectal prolapse measuring > 5 cm. The patient had no significant medical history.

The patient had no problem regarding surgical tolerance and had a history of undergoing laparoscopic anterior suture rectopexy without sigmoid resection under general anesthesia for complete rectal prolapse one year prior. The postoperative course was uneventful, and the patient was discharged 1 week postoperatively. However, her dementia worsened (Hasegawa’s dementia scale: 5/30 points) after the operation, and moderate-to-severe aortic valve stenosis was first diagnosed with heart failure 6 months after the operation. The ejection fraction was 65.4%, and echocardiography revealed diffuse thickening of the left ventricular wall.

Nine months after the initial surgery, she experienced a recurrence of complete rectal prolapse measuring approximately 5 cm. Considering the coexistence of advanced age, severe dementia, and aortic valve stenosis, surgery under general anesthesia was not indicated. Perineal stapled prolapse resection in combination with the Thiersch operation was planned because of its minimal invasiveness and shortened hospital stay. The procedure was performed by a team of two surgeons in the jackknife position under spinal anesthesia. The jackknife position was selected instead of the lithotomy position due to serious hip joint contracture.

**Fig. 1:**
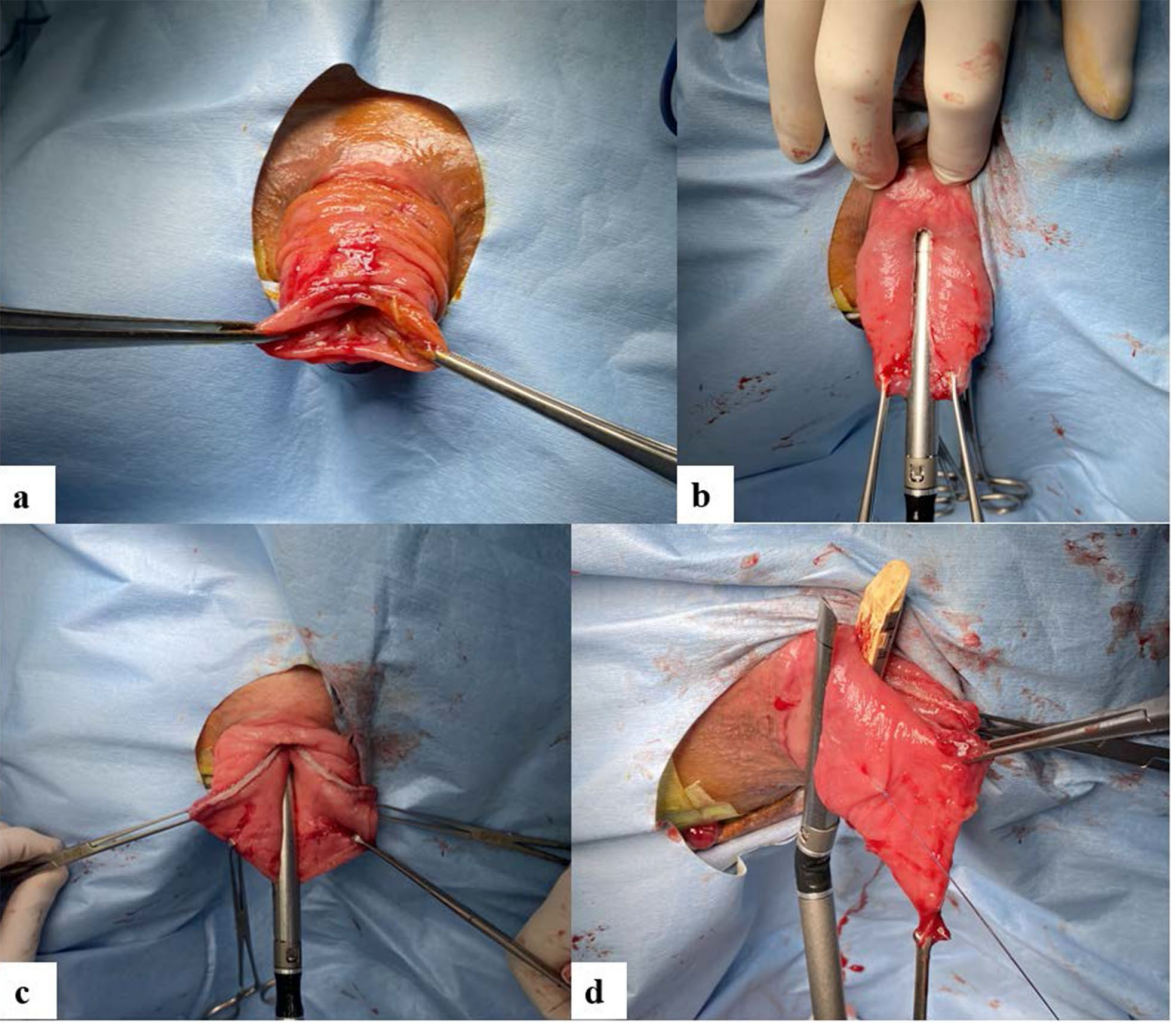
**a** A 5-cm prolapse of the rectum. **b** The prolapse of posterior wall was cut along the long-axis direction. **c** The prolapse of anterior wall was cut along the long-axis direction. **d** The prolapse was resected along the short-axis direction with four linear staplers

**Fig. 2:**
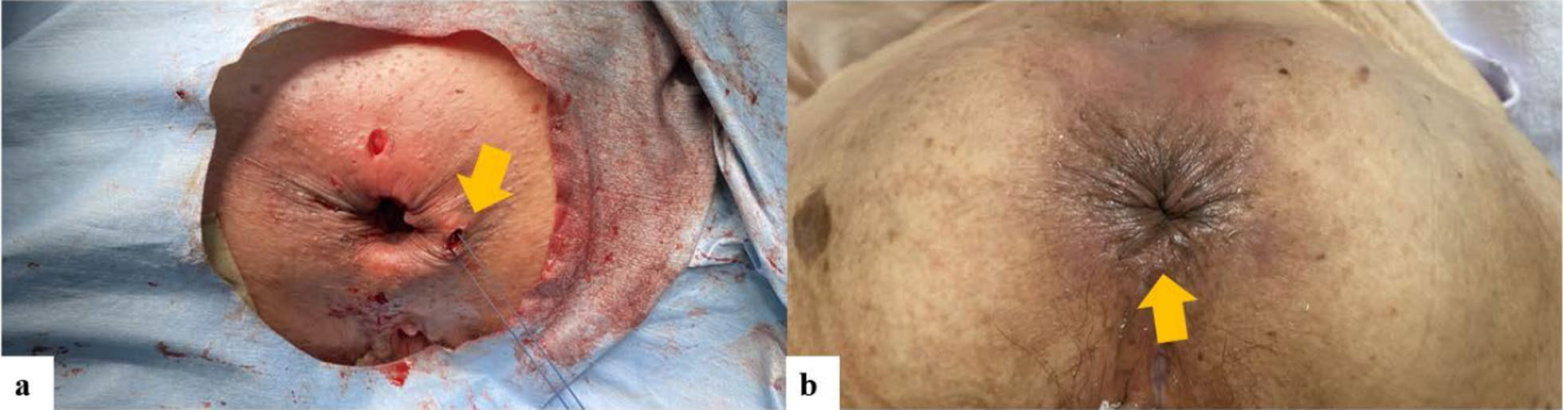
**a** The Thiersch operation was additionally performed with 1-0 nylon thread 1 cm away from the anal verge after perineal stapled prolapse resection. **b** Anal findings 2 months after surgery

The prolapse was completely pulled out and fixed using Allis clamps (Fig. 1a), then cut along its long axis with three linear staplers (× 2 cartridges in the 0 o'clock direction, × 1 cartridge in the 6 o'clock direction) (Powered Echelon Flex gold 60 mm) (Fig. 1b, c), and resected along its short-axis direction with four linear staplers (Fig. 1d). The cross-section of the linear staples was reinforced with 3-0 Vicryl suturing. After rectal resection, the Thiersch operation using 1-0 nylon thread 1 cm away from the anal verge was additionally performed (Fig. 2a). The operative time was 24 min and the intraoperative blood loss was 1 mL. The postoperative course was uneventful, and the patient was discharged two days postoperatively without any postoperative complications. Three months after the operation, no recurrence was observed and defecation function was good with improvements of Wexner score (18 → 5) (Fig. 2b).

## Discussion

Several mechanisms have been reported for rectal prolapse, including a spectrum of coexisting anatomic abnormalities, such as diastasis of the levator ani muscle, an abnormally deep cul-de-sac, a redundant sigmoid colon, a patulous anal sphincter, and loss or attenuation of the rectal sacral attachments [4]. The choice of treatment is important because the involvement of the pathogenesis varies from case to case.

If patient surgical tolerance is sufficient, anterior or posterior rectopexy is recommended for complete rectal prolapses measuring ≥ 5 cm accompanied by prolapse of the rectosigmoid junction [3].

Conventionally, the Delorme procedure or the Altemeier procedure has been recommended as a perineal operative option for rectal prolapse in patients with poor general condition. Previous studies reported that recurrence rates of the Altemeier procedure were within the range of 16 to 30% [5–7], and that of the Delorme procedure was within the range of 10 to 15% [7–9]. According to guidelines [3], the Altemeier procedure is appropriate for patients with a long (> 5 cm) full-thickness rectal prolapse. However, the Altemeier procedure requires transanal excision and hand-sewn anastomosis of the colon. Furthermore, the surgical technique is complicated and requires sufficient experience of the surgeons. Approximately 9–22% of patients experience complications, such as anastomotic leakage, anastomotic stenosis, pelvic hematoma, sigmoid colon perforation, pararectal abscess, and rectal–vaginal fistula. In particular, stoma creation may be required for severe anastomotic complications [5, 10]. Moreover, in cases of relapse rectal prolapse after the first Altemeier procedure, it is difficult to re-operate the same procedure because of severe adhesions caused by the primary operation.

In this case, the patient experienced relapsed rectal prolapse after anterior suture rectopexy. Since her surgical tolerance was decreased due to age and comorbidity (aortic valve stenosis, dementia), a lower invasive procedure and shorter operation time and postoperative hospital stay were necessary. Therefore, we performed perineal stapled prolapse resection in combination with the Thiersch operation.

Perineal stapled prolapse resection is a procedure that was first reported by Scherer et al. in 2008 and is known as a simple technique with a low complication rate [11]. Scherer et al. reported that, in 14 of 15 patients (93%), perineal stapled prolapse resection was successfully performed without complications, and the median operating time was 33 min (range: 22–52 min) [11]. Fan et al. reported that, in a systematic review of 408 patients who underwent perineal stapled prolapse resection, bleeding was the most common complication, and the total complication rate was 14.5% (51/350) [12]. However, in a recent systematic review, the median incidence of recurrence was 13.9% for perineal stapled prolapse resection, which was higher than that of the Altemeier procedure (11.4%) [10].

In this case, the pelvic floor muscles were weakened and the anal sphincter was relaxed with fecal incontinence preoperatively. We considered that performing the perineal stapled prolapse resection alone was not sufficient. Furthermore, in addition to the recurrence of rectal prolapse, improvement in bowel function was also an important parameter for the postoperative effectiveness of the procedure [10].

In this case, the Thiersch operation was additionally performed to reinforce the anal sphincter and to improve fecal incontinence. The Thiersch operation [13, 14], which was first described in 1943, is a simple surgical procedure that encircles the anus with a thread or silver wire. The Thiersch operation has been widely used for rectal prolapse or fecal incontinence [13, 14]. However, the recurrence rate following the Thiersch operation alone for rectal prolapse is reported to 15–67% [15–17]. For this reason, the Thiersch operation is usually performed in addition to other surgical procedures. A modified Gant–Miwa–Thiersch procedure [18] and the Delorme–Thiersch operation [19] for rectal prolapse were reported with recurrence rates of 7.5% and 7.8%, respectively. Therefore, an additional Thiersch operation, among other procedures, is effective for both decreasing the incidence of recurrence for rectal prolapse and improving fecal incontinence. To our knowledge, there have been no reports of the combination of perineal stapled prolapse resection and the Thiersch operation for complete rectal prolapse.

Our modified technique of perineal stapled prolapse resection included resection of the prolapse using only linear staplers instead of curbed cutter devices. In the original study [11], the prolapse was first cut open at three o'clock with a linear stapler along its long-axis direction, and then resected along its short-axis direction with curbed cutter devices. The curbed cutter device has a staple line length of 40 mm; in contrast, the linear stapler (Powered Echelon Flex gold) has a staple line length of 60 mm. Considering the medical cost and risk of anastomotic leakage at cross-section, we selected the linear stapler because it uses fewer cartridges. Furthermore, since a curbed cutter device was not used for resection in the short-axis direction, we considered making the excision line straighter from the curve, and two long-axis cuts using three cartridges were performed on the anterior and posterior walls (at 0 o'clock and 6 o'clock).

An important aspect of this procedure is that the number of cartridges required for intestinal resection is not constant. Rather, it is dependent on the length and thickness of the prolapsed rectum. As the cross-section between the resection stump becomes fragile, with a subsequent risk of stapling failure [20], we believe that it is important to reinforce the cross-section with absorbable threads. In addition, it is thought that staple line bleeding can be avoided by performing stapling over time and applying compression. For the Thiersch operation, a nylon thread with a low risk of wound infection was used.

As for preoperative preparation, since rectal prolapse causes marked edema due to changes in osmotic pressure, edema, and erosion, it is important to fully reposition the rectum just before surgery in preparation for a secure excision in order to reduce the risk of stapling failure.

A principal advantage of this procedure is that it can be easily performed even for relapsed cases because of its simplicity and minimal invasiveness. Another advantage of our procedure is that it can be performed regardless of the length of the prolapsed tract.

We have no data to substantiate the long-term outcomes of this treatment. This case had a short follow-up period, and careful follow-up is required in the future. Another limitation of this procedure was the high medical cost of the stapling devices. However, taking into account the shortened operation time as well as the short length of hospital stay with perineal stapled prolapse resection compared to other procedures, the higher medical costs might be compensated. Furthermore, perineal stapled prolapse resection had a theoretical possibility of blood flow insufficiency in the residual anal intestinal tract due to resecting together with the mesentery. However, but there have been no reports of this complication in past systematic reviews [10]. Further case accumulations are necessary to estimate the safety of this procedure.

## Conclusions

In conclusion, perineal stapled prolapse resection in combination with the Thiersch operation could be a useful option for patients with relapsed rectal prolapse and with poor general condition who are not indicated for other surgical procedures.

## Data Availability

Data sharing is not applicable to this article as no datasets were generated or analyzed during the current study.
